# HHLA2 predicts better survival and exhibits inhibited proliferation in epithelial ovarian cancer

**DOI:** 10.1186/s12935-021-01930-y

**Published:** 2021-05-07

**Authors:** Guocai Xu, Yuanyuan Shi, Xiaoting Ling, Dongyan Wang, Yunyun Liu, Huaiwu Lu, Yongpai Peng, Bingzhong Zhang

**Affiliations:** Department of Gynecologic Oncology, Sun Yat-Sen Memorial Hospital, Sun Yat-Sen University, Yingfeng Road No.33, Haizhu District, Guangzhou, Guangdong China

**Keywords:** HHLA2, Immune checkpoint, Prognosis, CD8^+^ tumour-infiltrating lymphocyte, Ovarian cancer

## Abstract

**Purpose:**

The role of HHLA2, a new immune checkpoint ligand, is gradually being elucidated in various solid tumours. However, its role in ovarian cancer remains unclear; thus, its expression profile and clinical significance in ovarian cancer must be examined.

**Methods:**

We performed immunohistochemistry to examine HHLA2 expression in 64 ovarian cancer tissues and 16 normal ovarian tissues. The relationships between HHLA2 expression and clinicopathological features, prognosis, and CD8^+^ tumour-infiltrating lymphocytes (TILs) in patients were analysed. Additionally, the Cancer Cell Line Encyclopedia database was used to analyse the correlation between HHLA2 expression and PD-L1 or B7x expression. Furthermore, the biological function of HHLA2 in ovarian cancer cells was initially explored.

**Results:**

Only 17.2% of ovarian cancer patients showed HHLA2 expression, which was significantly associated with the differentiation of ovarian cancer cells (*p* = 0.027), and well-differentiated tumours expressed higher levels of HHLA2. The density of CD8^+^ TIL was associated with increased HHLA2 expression (*p* = 0.017), and the CD8^+^ TIL count was higher in the HHLA2-positive group than that in the HHLA2-negative group (*p* = 0.023). Moreover, multivariate analysis identified HHLA2 expression as an independent prognostic factor that predicted improved survival (*p* = 0.049; HR = 0.156; 95% CI = 0.025–0.992). Additionally, we also found that overexpressing HHLA2 inhibited the proliferation of ovarian cancer cells.

**Conclusion:**

HHLA2 is associated with tumour differentiation and high CD8^+^ TIL levels; and predicts improved survival in ovarian cancer. Along with previously reported findings that HHLA2 behaves as a co-stimulatory ligand, our study suggests that the loss of HHLA2 may contribute to the immunosuppressive microenvironment and progression of ovarian cancer.

## Introduction

Ovarian cancer is a gynaecologic malignant disease, and more than 80% of patients with ovarian cancer present with advanced disease during diagnosis. The overall 5-year survival rate of ovarian cancer is < 30% [1]. According to the National Comprehensive Cancer Network (NCCN) guidelines, surgery and postoperative platinum-based chemotherapy are the primary treatments for ovarian cancer. However, most patients will experience multiple recurrences, most of whom would die from platinum-resistant disease [2]. Thus, identifying new treatment strategies remains an urgent need for ovarian cancer therapy. In recent years, anti-tumour immunotherapy has taken centre stage in mainstream oncology owing to the durable response rates in patients with multiple types of cancer [3].

Ovarian cancer has been recognized as a target for immunotherapy [4]. However, the immunosuppressive tumour microenvironment, with multiple components and pathways inhibiting effective tumour-targeted immune responses, is the main obstacle to the successful deployment of cancer immunotherapy for ovarian cancer patients [5]. Thus, there is great potential in targeting these mechanisms of immunosuppression to reverse them and create an environment that encourages anti-tumour immune responses. Immune checkpoint proteins are critical molecules that either activate or inhibit T-cell function [6]. The expression of inhibitory immune checkpoint ligands on cancer cells is one mechanism by which these cells evade the immune response [7]. Therefore, immune checkpoint therapy, which targets regulatory pathways in T cells, can restore the function of T cells and cause tumour regression. Inhibitors of immune checkpoints, such as antibodies against cytotoxic T lymphocyte-associated antigen 4 (CTLA4) and programmed death ligand-1/programmed death-1 (PD-L1/PD-1) molecules, can release the inhibited immune function of T cells and make the tumour free from immune tolerance, killing tumour cells [3]. Clinical trials of the above checkpoint inhibitors demonstrated that anti-tumour immunity could be augmented and improve overall survival in ovarian cancer [4]. Although the initial results of checkpoint blockade in ovarian cancer are promising, the anti-tumour efficacy remains limited. Potential mechanisms underlying the modest anti-tumour efficacy include the presence of multiple co-inhibitory ligands or receptors on cancer cells or T cells infiltrating tumours, compensatory upregulation of multiple immune checkpoints and other immunosuppressive mechanisms (e.g., Indoleamine 2,3-dioxygenase (IDO) and TGF-β) [5]. Therefore, monotherapy via checkpoint blockade may not be sufficient to generate strong anti-tumour responses. Identifying new immune targets and combination or alternative treatment strategies is warranted [3].

The newly discovered immune checkpoint molecule, human endogenous retrovirus-H long terminal repeat-associating protein 2 (HHLA2), may shed some light in this direction. HHLA2, with alternative names of B7-H5 and B7H7, is a new member of the B7 family that modulates T-cell function, analogous to PD-L1, PD-L2, B7-H3, and B7x [8]. In the immune system, HHLA2 is constitutively expressed by human monocytes and induces B cells [8], binding to receptors on T cells, B cells, and antigen-presenting cells. HHLA2 exerts both co-inhibitory and co-stimulatory functions [9–12]. As a T-cell co-inhibitor, HHLA2 suppresses the proliferation and cytokine production of both human CD4^+^ and CD8^+^ T cells [9, 11]. Additionally, the HHLA2/TMIGD2 interaction co-stimulates human T-cell growth and cytokine production via an AKT-dependent signalling cascade [10]. When expressed on antigen-presenting cells, HHLA2 stimulates T-cell proliferation and cytokine secretion via the transmembrane and immunoglobulin domain-containing 2 (TMIGD2) receptor and localises on naïve T cells. However, TMIGD2 expression is gradually lost as T cells undergo activation, leading to the inhibition of T-cell function through another unknown receptor [13].

HHLA2 protein expression is limited in normal human tissue, being restricted primarily to epithelial cells in the intestines, breast, and placenta [14]. In contrast, HHLA2 is highly expressed in most malignant tissues, including breast, lung, thyroid, and melanoma [8]. Interestingly, multiple researchers have pointed to the double-edge sword properties of HHLA2 in different tumour tissues. In breast cancer [8], clear cell renal cell carcinoma [15], colorectal cancer [16], and osteosarcoma [17], high HHLA2 expression is associated with invasive tumour behaviour and worse clinical outcomes. In contrast, in gastric cancer, patients with high HHLA2 mRNA levels in peripheral blood have a higher 5-year survival rate than those with low HHLA2 levels, and HHLA2 mRNA levels have a significant negative association with invasive tumour behaviours [18]. In pancreatic ductal adenocarcinoma, HHLA2 is associated with better survival rates and acts as a co-stimulatory ligand [12, 19]. Therefore, HHLA2 may serve as a new therapeutic target for tumour immune checkpoints in addition to CTLA-4, PD-1, and PD-L1.

To our best knowledge, no study on HHLA2 expression and function in ovarian cancer have been reported to date. Our present study examined the expression of HHLA2 in ovarian cancer and analysed its relationship with clinicopathologic features and prognosis to further explore its role in ovarian cancer, providing a primary foundation for the discovery of new immunotherapeutic targets for ovarian cancer.

## Materials and methods

### Patients and tissue samples

This study was approved by the Ethics Committee of Sun Yat-sen Memorial Hospital, Sun Yat-sen University, and informed consent was obtained from the patients. In total, 64 patients (age range 18–79 years; mean age, 52 ± 10 years) with epithelial ovarian cancer who had undergone surgical resection at Sun Yat-sen Memorial Hospital between 2007 and 2011 were enrolled in this study. All patients were treatment-naïve before surgery. Consecutive sections were cut from paraffin-embedded tissue specimens for immunohistochemical staining for HHLA2 expression and CD8^+^ T cells.

### Immunohistochemistry

Formalin-fixed epithelial ovarian cancer tissue and human placental villus tissue sections were deparaffinised, followed by antigen retrieval treatment using sodium citrate (10 mM, pH 6.0). Endogenous peroxidase activity was blocked via incubation in 3% H_2_O_2_ for 10 min. Goat serum (Beyotime, Shanghai, China) was used to block nonspecific staining. A mouse anti-human HHLA2 mAb (clone 566.1; IgG1) was used at a concentration of 4 µg/ml (dilution of 1: 500), and a rabbit anti-human CD8 mAb (Zhong Shan Golden Bridge Biotech, Beijing, China) was used at a dilution of 1:100 with overnight incubation. The HHLA2 antibodies produced and donated by Dr. Xingxing Zang were validated by previously published studies [8]. The Dako REAL Envision Detection System/HRP (Dako Corporation, Carpentaria, California, USA) was used to detect horseradish peroxidase (HRP)-conjugated rabbit/mouse universal secondary antibody followed by 3,3′-diaminobenzidine chromogen and haematoxylin nuclear counterstaining. The villus tissue of the placenta was selected as a positive control. Scoring was performed by two independent investigators. The results of HHLA2 staining were recorded as the tumour proportion score [20]. “Negative” indicated 0% HHLA2 staining, and “positive” indicated > 0% HHLA2 staining. Cells that were positively stained for CD8 were manually counted in at least five separate high-power fields under × 200 magnification, and the CD8^+^ T-cell density was calculated using the average count of positively stained cells.

### Oncomine data acquisition and analysis

We analyzed the expression of HHLA2 in ovarian cancer and normal ovarian tissue using the Oncomine database. The website of Oncomine is https://www.oncomine.org/resource/login.html. The TCGA data set was obtained from the Oncomine database. The data set included HHLA2 mRNA expression in 586 Ovarian Serous Cystadenocarcinoma tissue and 8 normal ovarian tissue. The HHLA2 mRNA expression level in ovarian cancer tissue and normal ovary tissue was compared using T test. P < 0.05 indicated statistical significance.

### Cancer Cell Line Encyclopedia (CCLE) data acquisition and analysis

To elucidate the correlation between HHLA2 and other immune checkpoints, The CCLE database was used for mRNA data collection for HHLA2, PD-L1, and B7x in ovarian cancer. The website of CCLE is https://portals.broadinstitute.org/ccle/about.

By inputting the name of the gene in the website, we can obtain the mRNA expression level of the gene in different tumour cell lines. The correlation of HHLA2 expression with that of PD-L1 and B7x was analysed using Spearman’s test. P < 0.05 indicated statistical significance.

### Cell lines

SK-OV-3, HO-8910, A2780, OVCAR-3, and ES-2 human ovarian cancer cell lines were provided by Dr. Limin Zheng. The PEO1 human ovarian cancer cell line was a gift from Dr. Qien Wang. All the above cell lines except A2780 and ES-2 cell lines are derived from ovarian serous adenocarcinoma, which accounts for most ovarian cancers. We used the OVCAR-3 and PEO1 cell lines to establish stable cell lines overexpressing HHLA2 based on their lower expressions of HHLA2 we found. Lentivirus carrying the HHLA2 sequence was used to transfect OVCAR-3 and PEO1 cell lines for 8–12 h. After transfection, the RPMI-1640 medium was replaced, and transfection was continued for 48 h. Next, the cells were selected with 2 µg/ml of puromycin to establish the stably transfected cell lines OVCAR-3-HHLA2-Flag and PEO1-HHLA2-Flag with high levels of HHLA2 expression. OVCAR-3 and OVCAR-3-HHLA2-Flag cells were maintained in RPMI-1640 containing 20% foetal bovine serum (FBS) (Gibco, California, USA). The other cell lines were maintained in RPMI-1640 containing 10% FBS.

### Western blot analysis

Proteins were extracted using radioimmunoprecipitation assay (RIPA) buffer (Beyotime, Shanghai, China) containing 1% phenylmethanesulphonyl fluoride. Forty-microgram protein samples were subjected to 10% SDS-PAGE and then were transferred to polyvinylidene fluoride membranes after separation. After blocking using 5% skim milk, the membranes were incubated with anti-HHLA2 antibody (ab214327; Abcam, Cambridge, MA, USA) overnight at 4 °C and incubated with HRP-conjugated secondary antibody for 2 h at room temperature. GAPDH (5174S; Cell Signaling Technology, Ipswich, MA, USA) was used as an internal control. Signals were detected by enhanced chemiluminescence (ECL) (Thermo Scientific, Waltham, MA, USA).

### Total RNA extraction and qRT–PCR

The cells were dissociated and resuspended in 1 ml of TRIzol reagent (Thermo, Waltham, USA), and total RNA was extracted according to the manufacturer’s instructions. In total, 500 ng of total RNA was used for cDNA synthesis using ReverTra Ace® qPCR RT Master Mix with gDNA remover (Toyobo, Osaka, Japan). For qPCR, PCR mixtures were prepared using an AceQ® qPCR SYBR® Green Master Mix kit (Vazyme Biotech, Nanjing, China). The relative expression levels were analysed using the 2^−ΔΔCT^ method. HHLA2 primer sequences were as follows:

Forward: 5′-TACAAAGGCAGTGACCATTTGG-3′,

Reverse: 5′-AGGTGTAAATTCCTTCGTCCAGA-3′.

### 3-(4,5-dimethyl-2-thiazolyl)-2,5-diphenyl-2H-tetrazolium bromide (MTT) assay

Cell viability was detected using the MTT assay. OVCAR-3 and PEO1 parental cell lines and their HHLA2-overexpressing cell lines were seeded in 96-well plates (1000 cells/well) and allowed to adhere overnight. Twenty-four hours after seeding, the cells were incubated with MTT (Sangon Biotech, Shanghai, China) for 4 h at 37ºC, after which the culture medium was removed, 150 μl of DMSO (Sangon Biotech, Shanghai, China) was added, and the absorbance values were measured at 570 nm. Next, the absorbance at 570 nm was measured in sequence for the subsequent 4 days. We defined the value for the first day as the control. The relative survival was normalised to the control level after background subtraction.

### 5-Ethynyl-2′-deoxyuridine (EdU) staining

Cell proliferation was evaluated using the EdU assay. OVCAR-3, OVCAR-3-HHLA2, PEO1, and PEO1-HHLA2 cells were plated at a density of 4 × 10^4^ cells/ml per well in 6-well plates and cultured for 48 h. Next, the cells were incubated with 10 μM EdU for 1.5 h, fixed with 4% paraformaldehyde for 15 min, and stained using the BeyoClick™ EdU-594 proliferation assay kit (Beyotime, Shanghai, China) according to the manufacturer’s instructions. Images were acquired under an inverted fluorescence microscope (Carl Zeiss AG, Oberkochen, Germany). Cells with red staining were considered EdU positive.

### Statistical analysis

All statistical analyses were performed using SPSS version 21.0 software (SPSS Inc., Chicago, IL, USA). χ^2^ test, the Mann–Whitney U test, and the Kruskal–Wallis H test were used to investigate the associations of HHLA2 expression with the pathological features of patients and CD8^+^ T-cell infiltration. Survival analyses were conducted using the Kaplan–Meier method and Cox proportional hazards model. Student’s *t*-test was used for statistical analysis for the results of qPCR, MTT and EdU staining assays. A* p* value < 0.05 denoted statistical significance.

## Results

### Clinical and pathological features of patients and HHLA2 expression in ovarian cancer and normal ovarian tissue

**Fig. 1:**
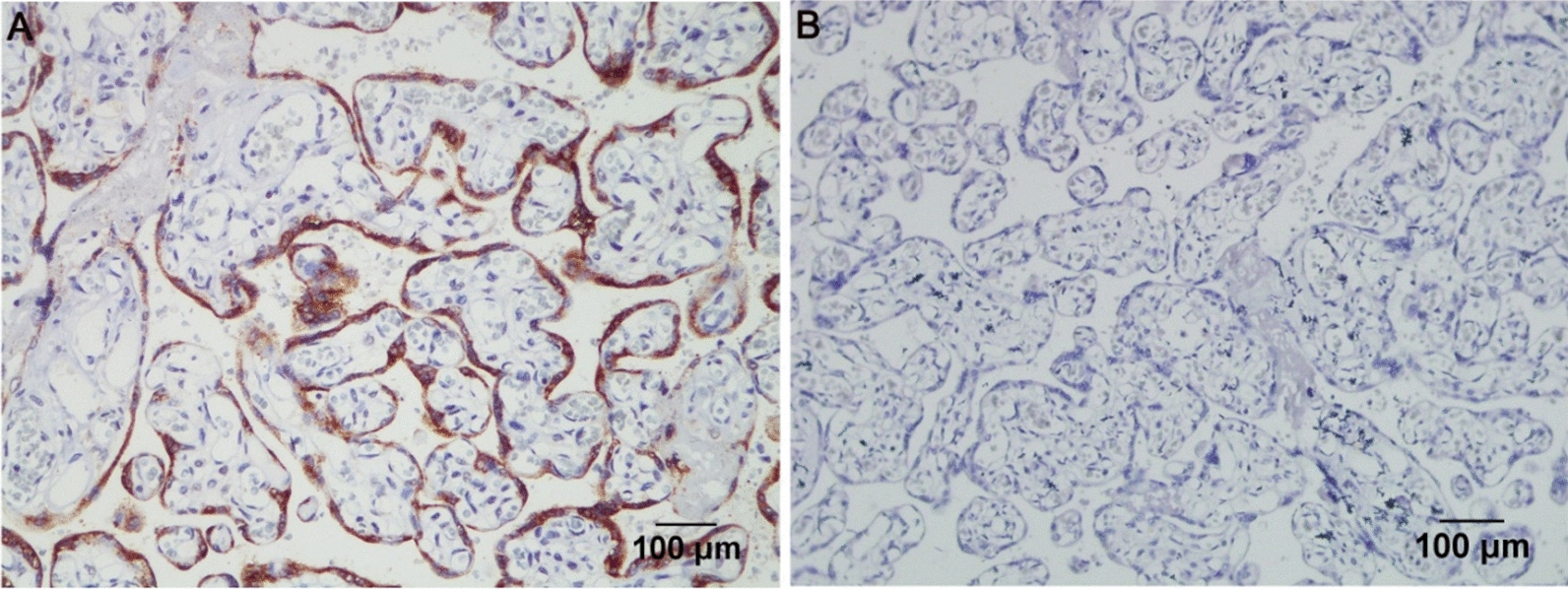
Detection of HHLA2 expression in placenta as a positive control. **a** A mouse anti-human HHLA2 mAb (clone 566.1, IgG1) was used to stain placenta as positive control; **b** A mouse anti-human isotype IgG1 was applied for negative control. Magnification × 200; Scale bar: 100 μm

**Fig. 2:**
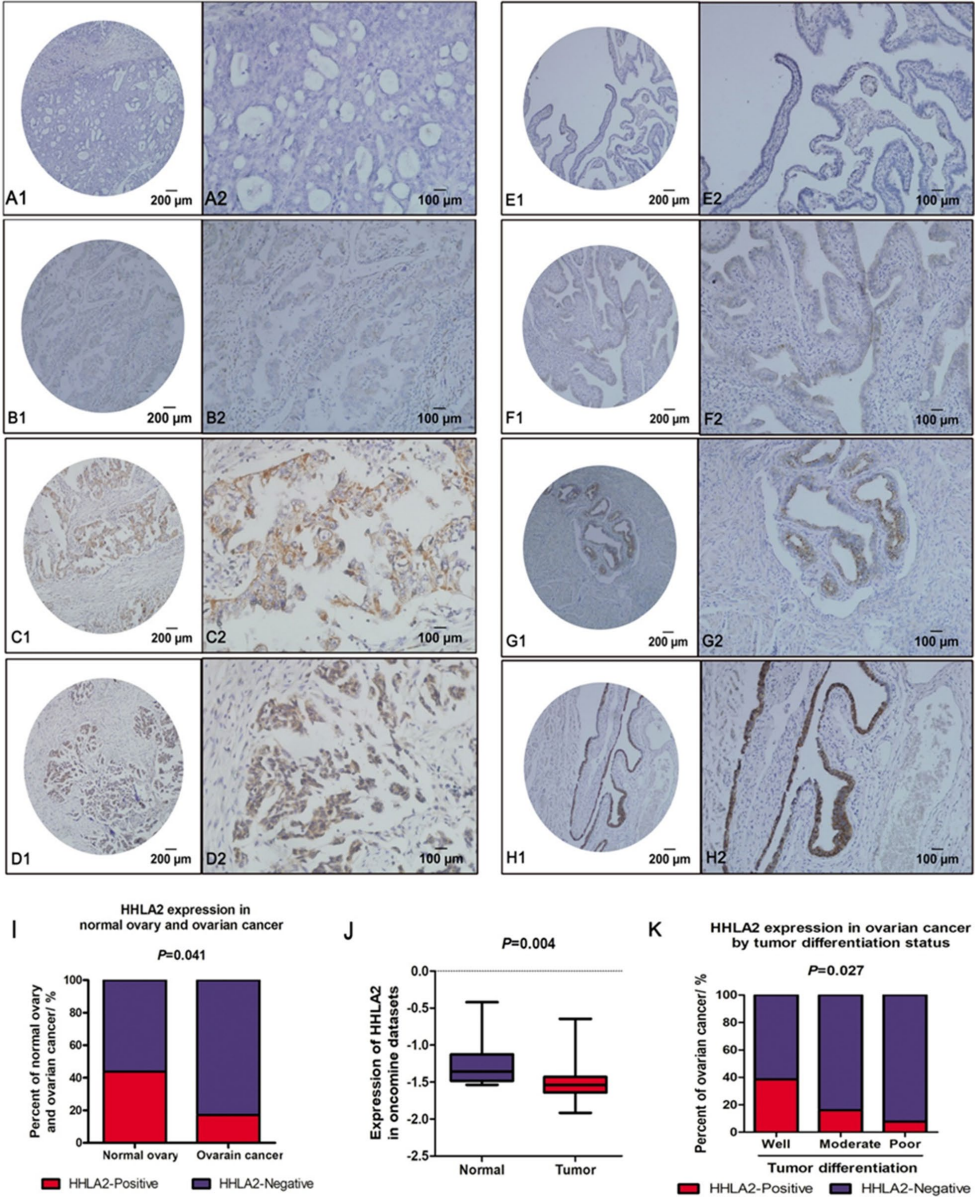
HHLA2 expression in ovarian cancer and normal ovary tissue. HHLA2 was stained with anti-HHLA2 antibody, and HHLA2-positive staining was detected in a membranous and cytoplasmic pattern in ovarian cancer and normal ovarian tissue epithelium. **a**–**d** represented epithelial ovarian cancer tissue obtained from 64 patients with ovarian cancer who had undergo surgical resection between 2007 and 2011. All patients were treatment-naïve before surgery. The level of HHLA2 expression was graded as intensity of IHC staining in ovarian cancer: A1-A2, absent; B1-B2, weak; C1-C2, moderate; D1-D2, strong. **e**–**h** The level of HHLA2 expression was graded as intensity of IHC staining in normal ovarian tissue epithelium: E1-E2, absent; F1-F2, weak; G1-G2, moderate; H1-H2, strong. Magnification: × 100 (A1–H1); × 200 (A2–H2). **i** The positive rate of HHLA2 expression was higher in normal ovary tissue than that in ovarian cancer. The red column represented the HHLA2 positive group, the blue column represented the HHLA2 negative group. The difference between them is significant.(*p* < 0.05). **j** Expression of HHLA2 was frequently decreased in 585 ovarian cancer tissues (Tumor) compared with 8 normal ovary tissue (Normal) samples in the oncomine dataset. **k** The proportion of HHLA2-positive patients was gradually increased with the improvement of tumour differentiation degree. HHLA2 tended to be expressed in well-differentiated ovarian tumour. The red column represented the HHLA2 positive group, the blue column represented the HHLA2 negative group. The difference between them is significant. (*p* < 0.05)

According to previous studies, HHLA2 protein is expressed in the villi of the placenta. Therefore, the villus tissue of the placenta was selected as a positive control in this study to test the effectiveness and specificity of the antibody. High HHLA2 staining was observed in the placenta compared with that in the negative control group, indicating the efficacy and specificity of the antibody used in the current research (Fig. 1). HHLA2 was mainly expressed in the cytoplasm and membrane in ovarian cancer cells. The level of HHLA2 protein in epithelial ovarian cancer tissue obtained from 64 patients with ovarian cancer who had undergo surgical resection between 2007 and 2011 was examined, and it was graded as follows: absent staining, weak staining, moderate staining, and strong staining (Fig. 2a–d). However, HHLA2 was not expressed in most ovarian cancer tissues, and its expression was only observed in a few ovarian cancer tissues. The positive rate of HHLA2 expression in ovarian cancer tissues in the current study was 17.2% (11/64), which was considered a low percentage compared with 61 ~ 66% in NSCLC [11, 21], 47.6% in colorectal cancer [16], 50% in clear cell renal cell carcinoma [15], and 56% in triple-negative breast cancer [8].

There is a constraint in obtaining paired normal adjacent tissue in ovarian cancer patients because most patients were diagnosed with advanced disease, making it almost impossible to obtain normal tissue samples within the cancerous tissue vicinity. For comparison, normal ovarian tissues were collected from patients with non-ovarian lesions who had undergone hysterectomy and bilateral adnexectomy (n = 16). We further analysed HHLA2 expression in these ovary tissues, grading the expression as four levels (Fig. 2e–h). We found that HHLA2 was highly expressed in normal ovarian epithelium with a positive rate of 43.75% (7/16), which was significantly higher than that in ovarian cancer tissues (*p* = 0.041, Fig. 2i), indicating the association of reduced HHLA2 expression with pathological changes during tumourigenesis. The HHLA2 expression profile in the Oncomine dataset also supports our result that the expression level in the normal ovary is significantly higher than that in ovarian cancer (*p* = 0.004, Fig. 2j).

### Associations of HHLA2 expression with the clinicopathologic characteristics in ovarian cancer

All tissue specimens were analysed by clinically certified pathologists who were blinded to the study, and the pathological features of the patients are shown in Table 1.

**Table 1:** Clinicopathological parameters of 64 ovarian cancer patients

**Characteristics**	**No**
Age, years	
≥ 50	42
< 50	22
Histological type	
Serous Adenocarcinoma	50
Mucinous Adenocarcinoma	2
Endometrioid Adenocarcinoma	6
Clear Cell Carcinoma	5
Transitional Cell Carcinoma	1
FIGO stage	
I	9
II	6
III	36
IV	13
Differentiation	
Poor	27
Moderate	24
Well	13
Ascites	
+	11
** −**	53
Vessel Invasion	
+	3
** −**	61
Lymph Node	
+	8
** −**	56
Platinum Sensitivity	
Sensitive	50
Resistance	14
Relapse	
+	27
** −**	37

To clarify the function and significance of HHLA2 in ovarian cancer, the correlations of HHLA2 expression with clinicopathologic characteristics, such as tumour stage, tumour differentiation, and histological type, were further analysed in the present study. An association was found between HHLA2 expression and the degree of differentiation of ovarian cancer (*p* = 0.027, Table 2). In poorly and moderately differentiated ovarian cancer, the proportions of HHLA2-positive patients were 7.69 and 16%, respectively; in well-differentiated patients, the proportion of HHLA2 positive patients was 38.46% (Fig. 2k). Our Study suggested that HHLA2 expression is higher in well-differentiated ovarian cancer than in poorly differentiated ones. No significant correlation was found between HHLA2 expression and the other clinicopathologic features (Table 2).

**Table 2:** Correlation of HHLA2 expression with clinicopathological parameters in ovarian cancer

Variable	Number of patients	HHLA2 Expression		
		+	−	*P* Value
Age				
≥ 50	42	7	35	1.000
< 50	22	4	18	
Histological type				
Serous adenocarcinoma	50	8	42	0.940
Others	14	3	11	
Differentiation				
Poor	26	2	24	**0.027**
Moderate	25	4	21	
Well	13	5	8	
FIGO Stage				
I	9	3	6	0.160
II	6	1	5	
III	36	6	30	
IV	13	1	12	
Metastasis				
+	51	8	43	0.532
−	13	3	10	
Ascites				
+	11	1	10	0.732
−	53	10	43	
Vessel Invasion				
+	3	0	3	1.000
−	61	11	50	
Lymph Node				
+	8	2	6	0.900
−	56	9	47	
Platinum Sensitivity				
Platinum sensitive	50	8	42	0.940
Platinum resistance	14	3	11	
Relapse				
+	27	4	23	0.925
−	37	7	30	
Serum CA125 Before Surgery				
< 35	4	1	3	0.539
≥ 35	60	10	50	
CA125				
+	37	5	32	0.148
−	5	2	5	
Unknown	19	4	16	
ER				
+	39	8	34	1.000
−	17	3	15	
Unknown	4	0	4	
PR				
+	33	6	29	1.000
−	23	5	20	
Unknown	4	0	4	

Bold value indicates* P* < 0.05

*ER*, Estrogen Receptor, *PR* Progesterone Receptor

### Prognostic value of HHLA2 protein expression in ovarian cancer

**Fig. 3:**
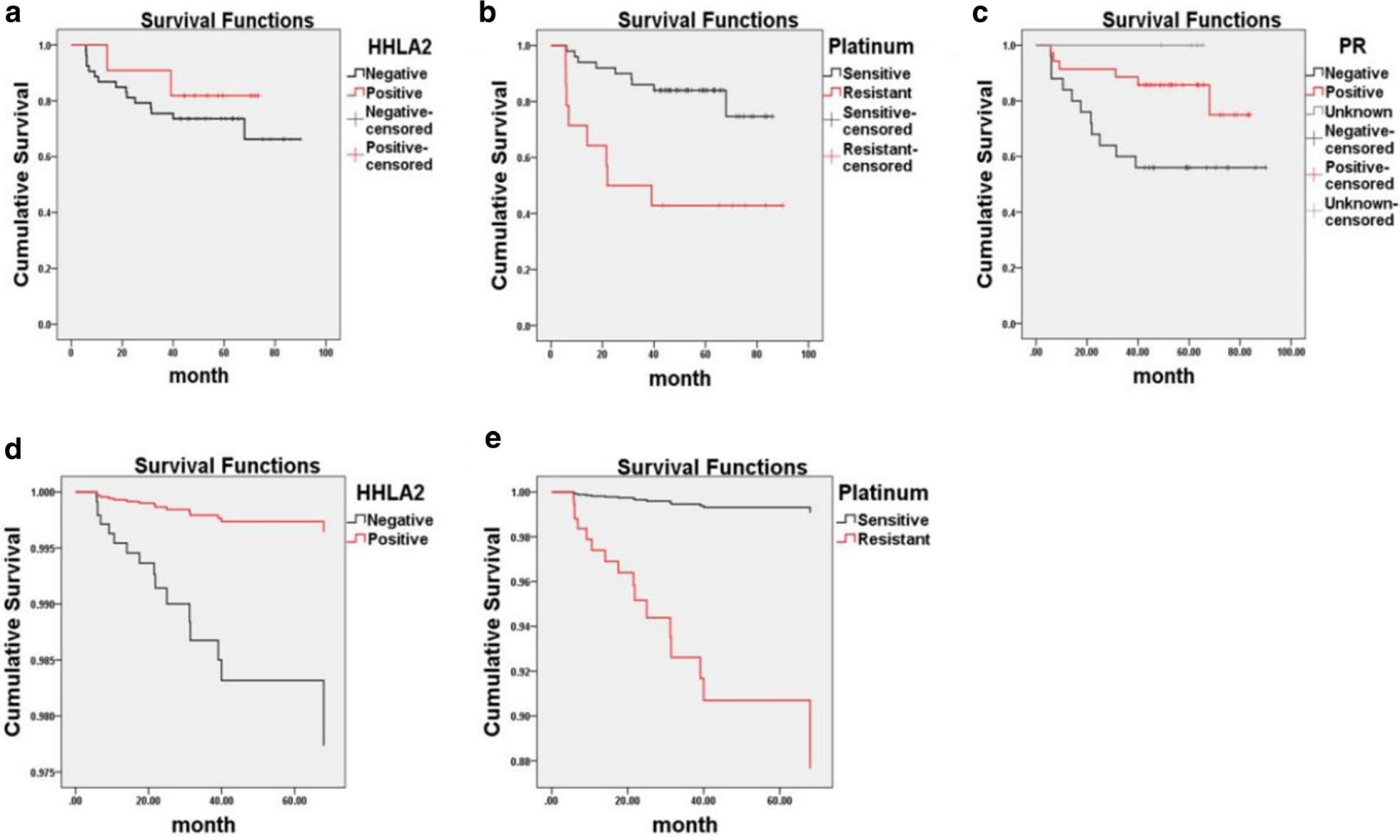
Kaplan–Meier survival curves and Cox proportional hazards regression model curves of ovarian cancer patients. Kaplan–Meier survival analysis showed that **a** HHLA2 positive patients were correlated with slightly superior overall survival rates (Breslow’s test, *p* = 0.474). **b** platinum-resistant patients had significantly shortened survival time compared to platinum-sensitive patients. (Breslow’s test, ***p < 0.001). **c** Progesterone receptor (PR) negative patients tended to have shorter survival than PR positive patients. **d**, **e** Cox proportional hazards regression model demonstrated that HHLA2 expression (*p = 0.049, HR = 0.156, 95% CI = 0.025–0.992) in cancer tissue and sensitivity to platinum chemotherapy (**p = 0.002, HR = 14.14, 95% CI = 2.632–75.916) were independent prognostic factors. Platinum-resistant patients and HHLA2-negative patients had significantly inferior overall survival rates with adjusting confounding factors

Kaplan–Meier survival analysis of HHLA2 expression and other clinicopathologic characteristics in patients with ovarian cancer was performed. HHLA2-positive patients showed better overall survival than the HHLA2-negative group, but without a significant difference (Breslow’s test; *p* = 0.474, Fig. 3a). Patients with platinum-sensitive tumours had a better prognosis than those with platinum-resistant tumours (Breslow’s test; *p* < 0.001, Fig. 3b). The 5-year cumulative survival rate of progesterone receptor (PR)-positive patients was also higher than that of progesterone receptor (PR)-negative patients (Breslow’s test; *p* = 0.02, Fig. 3c).

Univariate and multivariate Cox proportional hazards regression analyses were conducted to adjust for confounding factors and identify independent factors influencing the survival of patients with ovarian cancer (Table 3). In univariate analysis, fourteen clinicopathologic parameters potentially affecting survival were analysed, and sensitivity to platinum chemotherapy and PR expression in cancer tissues affected patient prognosis with unadjusted hazard ratios (HRs) of 4.32 [95% confidence interval (CI) = 1.652–11.294] and 0.328 (95% CI = 0.121–0.889), respectively. Other clinicopathologic features did not influence survival outcomes. In multivariate Cox regression analysis, along with the *p* values obtained in univariate analysis, the forward or backward models were used to screen the covariates with HR or regression coefficient β changes of at least 10% after the introduction or removal of covariates from the multivariate model, and the selected covariates were introduced into the equation [22]. Finally, HHLA2 expression in cancer tissue (*p* = 0.049; HR = 0.156; 95% CI = 0.025–0.992) and sensitivity to platinum chemotherapy (*p* = 0.002; HR = 14.14; 95% CI = 2.632–75.916) were identified as independent factors influencing the patient prognosis (Fig. 3d, e), and HHLA2 was an indicator of a better prognosis for ovarian cancer.

**Table 3:** Univariate and multivariate analysis of prognostic factors in ovarian cancer

Variable	Univariate Analysis			Multivariate Analysis		
	HR	*P* Value	95% CI	HR	*P* Value	95% CI
Expression of HHLA2	0.577	0.465	(0.132,2.523)	0.156	**0.049**	(0.025,0.992)
Positive *vs* Negative						
Age (years)	0.711	0.522	(0.250,2.019)			
< 50 vs ≥ 50						
Differentiation	0.53	0.399	(0.121,2.320)			
Poor and moderate *vs* well						
Pathologic type	0.439	0.275	(0.100,1.922)			
Serous adenocarcinoma vs other						
FIGO Stage	2.568	0.211	(0.586,11.248)			
I and II *vs* III and IV						
Ascites	0.275	0.211	(0.036,2.076)			
Positive *vs* Negative						
Vessel	1.618	0.641	(0.213,12.262)			
Positive *vs* Negative						
Lymph Node	1.763	0.376	(0.502,6.190)			
Positive *vs* Negative						
Relapse	1.142	0.789	(0.433,3.011)			
Relapse *vs* No Relapse						
Serum CA125 Before Surgery	22.49	0.469	(0.005,101,864.851)			
< 35 *vs* ≥ 35						
Platinum Sensitivity	4.32	**0.003**	(1.652,11.294)	14.14	**0.002**	(2.632,75.916)
Sensitive *vs* Resistant						
CA125	1.978	0.511	(0.259,15.137)			
Positive *vs* Negative						
ER	0.434	0.087	(0.167,1.128)			
Positive *vs* Negative						
PR	0.328	**0.028**	(0.121,0.889)			
Positive *vs* Negative						

Bold values indicate* P* < 0.05

*ER* Estrogen Receptor, *PR* Progesterone Receptor

### HHLA2 expression and CD8^+^ til density

**Fig. 4:**
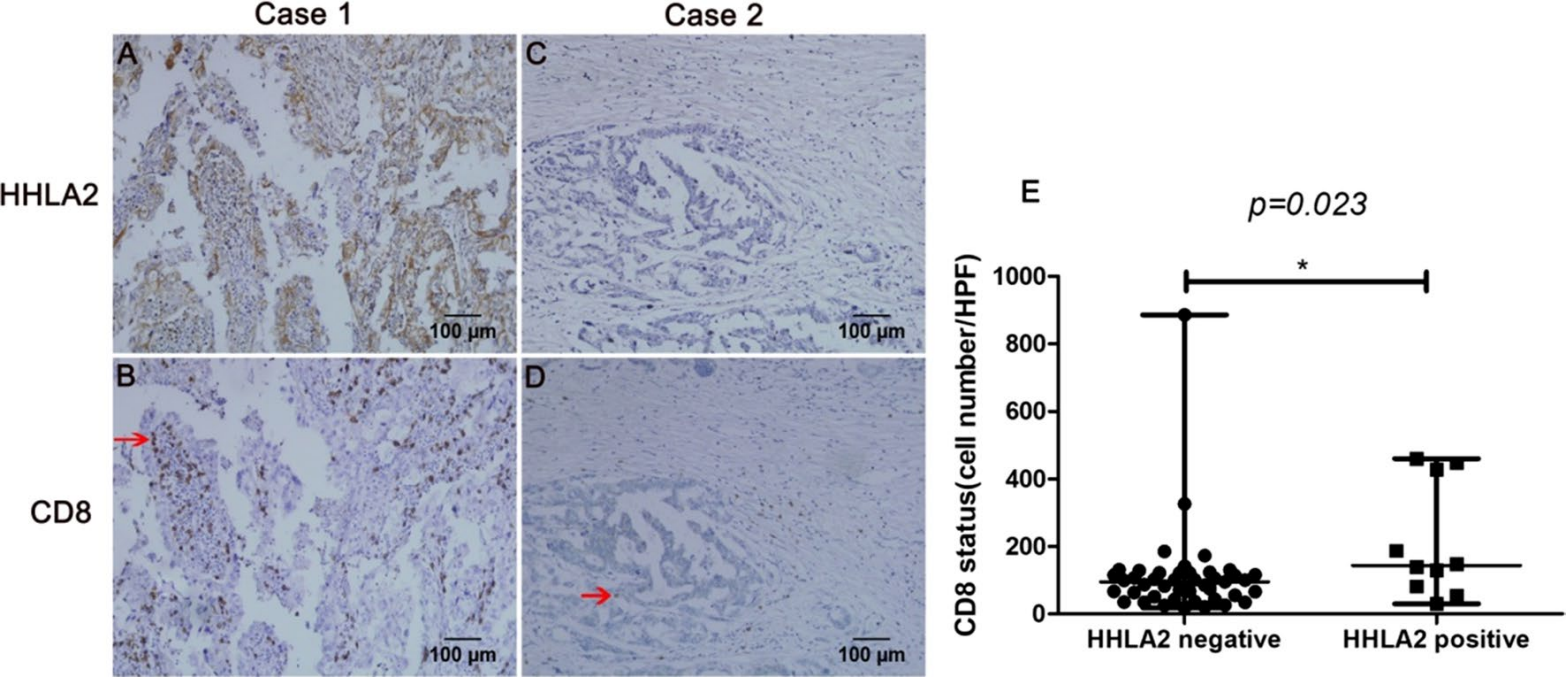
Association of HHLA2 expression in ovarian cancer cells and CD8^+^ TIL status. The HHLA2 positive cells and CD8 positive cells were stained by IHC. **a** HHLA2 positive and **c** HHLA2 negative staining on ovarian cancer cells. **b**, **d** CD8 staining on ovarian cancer tissue slides. The red arrowhead indicated CD8^+^T cells. Magnification: × 200. A, B and C, D were from the same tumour tissue respectively. **e** The average CD8^+^ TIL number was compared between patients with negative HHLA2 expression and positive HHLA2 expression

HHLA2 exerts its immunomodulatory effects via CD8^+^ T-cell proliferation and cytokine secretion [9, 10]. The correlation between HHLA2 expression and CD8^+^ T cells in ovarian cancer was evaluated in the present study. χ^2^ test revealed a significant association between HHLA2 expression and the CD8^+^ T-cell count in the tumour (*p* = 0.017, Table 4). Comparison of the CD8^+^ T-cell infiltration between HHLA2-positive (Fig. 4a, b) and -negative (Fig. 4c, d) patients illustrated that the CD8^+^ T-cell density was increased in patients with HHLA2-positive ovarian cancer. The difference in the CD8^+^ T-cell density between the HHLA2-positive and -negative group was significant (*p* = 0.023, Fig. 4e).

**Table 4:** Association between HHLA2 and CD8 TIL status

	HHLA2		*P*
	+	−	
CD8 status			
High(≥ 65)	9(90%)	19 (42.2%)	0.017
Low (< 65)	1(10%)	26 (57.8%)	

*TIL* Tumor-Infiltrating Lymphocyte

### Correlation between HHLA2 expression and that of immune checkpoint proteins PD-L1 and B7x in ovarian cancer

**Fig. 5:**
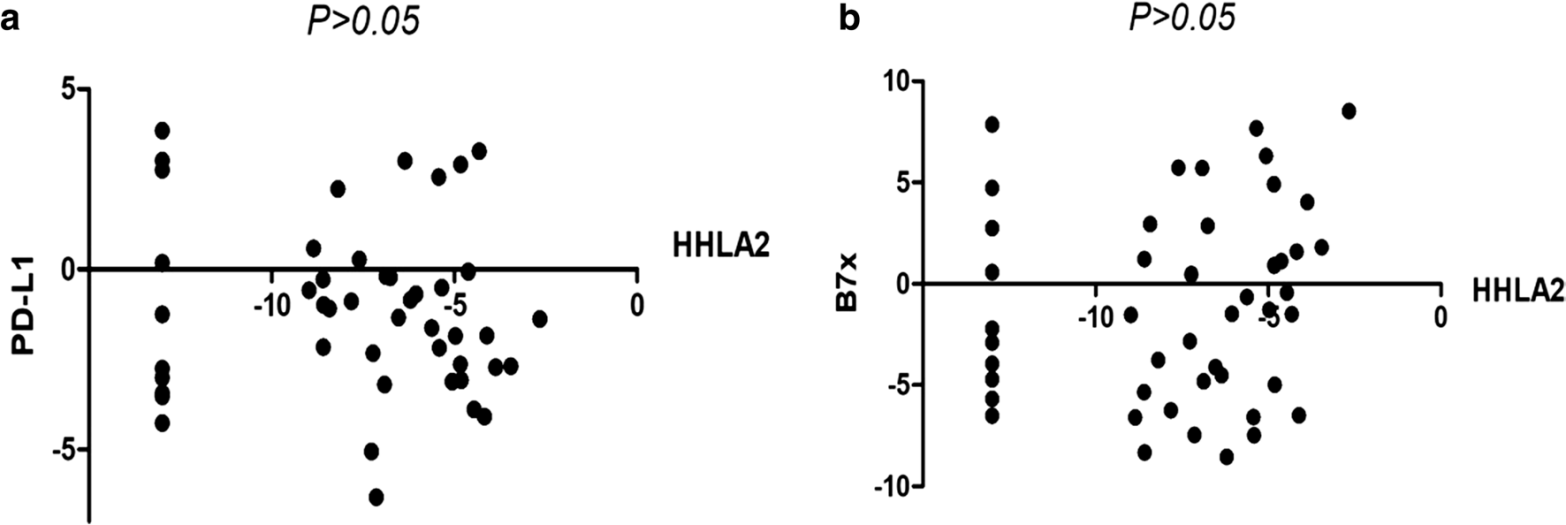
HHLA2 is not associated with PD-L1, B7x in ovarian cancer cells. **a** Correlation between the level of HHLA2 and PD-L1 markers in a panel of 47 breast cancer cell lines from CCLE database. **b** Correlation between the level of HHLA2 and B7x markers in a panel of 47 breast cancer cell lines from CCLE database

Previous studies have illustrated that HHLA2 is mainly expressed in PD-L1-negative lung cancer compared with that in PD-L1-positive lung cancer [11]. Additionally, HHLA2 was found to be co-expressed with B7x [11]. Therefore, to elucidate the relationships between HHLA2 expression and PD-L1 and B7x expression in ovarian cancer, the CCLE database was used to analyse the mRNA levels of HHLA2, PD-L1, and B7x in 47 ovarian cancer cell lines using Spearman’s correlation analysis. No correlation was found between HHLA2 expression and PD-L1 and B7x expression (Fig. 5a, b). Therefore, we speculate that HHLA2 was unlikely to be co-expressed with PD-L1 or B7x in ovarian cancer.

### HHLA2 inhibits the proliferation of ovarian cancer cells

Current research on HHLA2 has mainly focused on its regulation of the tumour microenvironment, particularly in the recruitment of TILs. However, studies of the tumour-intrinsic effect of HHLA2 signalling have not been reported. In the present study, the intrinsic function of HHLA2 in ovarian cancer cells was explored.

**Fig. 6:**
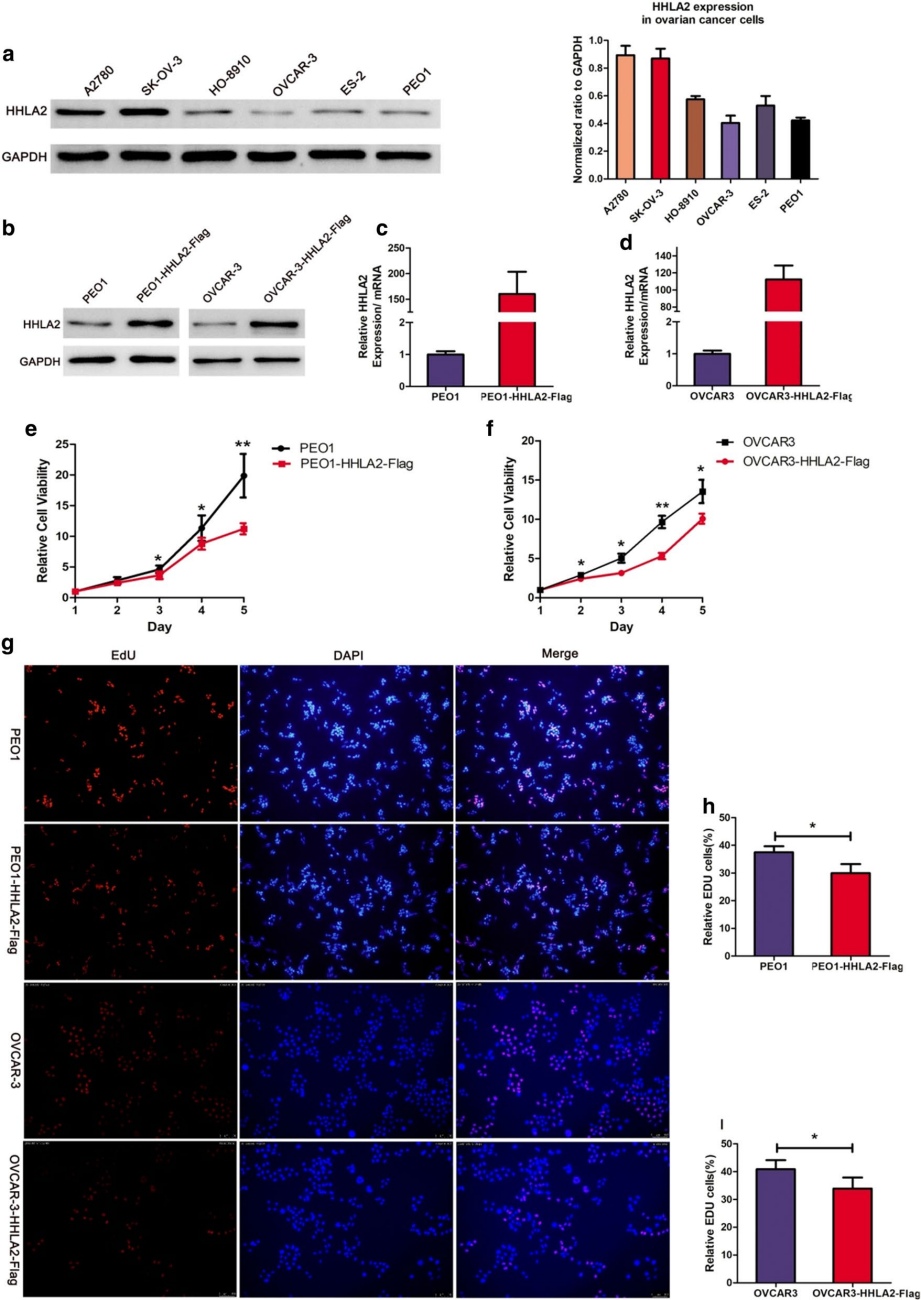
Upregulation of HHLA2 inhibits immune-independent ovarian cancer cells proliferation. **a** Western blotting of HHLA2 expression in ovarian cancer cell lines. The quantitative fold change in expression of HHLA2 normalized to GAPDH expression levels. The column on the right represented the relative expression level of HHLA2 in different ovarian cancer cell lines by Gray analysis. **b** Western blotting analysis of HHLA2 expression in PEO1 and OVCAR-3 cells and their HHLA2-overexpression counterparts. HHLA2-overexpression cells were transfected with lentivirus. GAPDH was detected as a loading control. **c** The expression level of HHLA2 in PEO1 and OVCAR-3 cells and their HHLA2-overexpressing counterparts were verified by qRT-PCR. ACTIN was detected as internal control. **e**, **f** Cell viability was evaluated by MTT assay at 1–5 days after cell seeded. The black curve represented the parental cell lines, the red curve represented the HHLA2-overexpression cell lines. It revealed that overexpression of HHLA2 significantly decreases the growth rate of PEO1 and OVCAR-3 cells(*p < 0.05, ** *p* < 0.01). **g** The EdU proliferation assay was performed in the HHLA2-overexpressing cell lines and their corresponding control groups. The cells with red fluorescence are in the S phase of mitosis, and the cells with blue fluorescence represent all of the cells. **h**, **i** Overexpression of HHLA2 decreased the mean proportion of proliferating ovarian cancer cells. The blue and the red column represented the proportion of proliferating cells in control and the HHLA2-overecpression group, respectively. **h**.* *p* = 0.027; i* *p* = 0.029. Three independent experiments were conducted for each assay

HHLA2 expression in ovarian cancer cell lines was initially measured, revealing that HHLA2 is highly expressed in A2780 and SK-OV-3 cells and weakly expressed in other cell lines (Fig. 6a). As a proof-of-concept, low HHLA2-expressing OVCAR-3 and PEO1 cells were selected to construct HHLA2-overexpressing cell lines to investigate the intrinsic biological function of HHLA2 in ovarian cancer (Fig. 6b–d). The MTT assay and EdU proliferation assays were conducted to determine the function of HHLA2 in ovarian cancer cells. Interestingly, the MTT assay revealed that HHLA2 overexpression significantly decreased the cell viability of OVCAR3 (from the 2nd day) and PEO1 (from the 3rd day) cells compared with that of the control groups (Fig. 6e, f). The EdU assay demonstrated that EdU incorporation was significantly decreased in the HHLA2-overexpressing groups compared with that in the control groups (Fig. 6g–i). The MTT and EdU assays both revealed that HHLA2 overexpression significantly reduced the proliferation of ovarian cancer cells. These results indicate a negative correlation between HHLA2 expression and tumour cell growth and proliferation.

## Discussion

Ovarian cancer is the most lethal of the gynaecologic malignancies. The main treatment for ovarian cancer is surgery and adjuvant chemotherapy. However, the 5-year survival rate of ovarian cancer has not benefited a lot from the improvement of above two measures [5]. New treatment strategies and paradigms are needed for these patients. In recent years, immunotherapies, particularly those targeting immune checkpoints, have been considered a new and effective strategy for ovarian cancer [23]. With the successful application of drugs targeting immune checkpoint and its receptor, such as PD-1 inhibitors in melanoma, researchers have also carried out clinical trials of using these drugs in ovarian cancer, but the effect is not ideal. The clinical study released in annual ASCO meeting reported that only 8 out of 75 patients achieved a partial response, and none had a complete response [24]. Some researchers have found that there are different immune checkpoint molecules and related pathways in different tumours and their immune environments. It is important to find and target these pathways, so as to remove the compensation caused by targeting a single pathway. As a newly discovered member of the B7 family, HHLA2 has gradually attracted researchers' attention.

HHLA2 has been described as having either co-inhibitory or co-stimulatory properties, depending on the malignancy type [14]. For example, a high HHLA2 expression level indicates a poor prognosis in osteosarcoma [17], colorectal cancer [16], and triple-negative breast cancer [8]. And some researchers have revealed that HHLA2 inhibits the proliferation and cytokine production of both human CD4^+^ T cells and CD8^+^ T cells, and functions as a T-cell co-inhibitory molecule [9, 11]. Thus, it indicates that high level of HHLA2 potentially plays an important role in tumour progression through immune suppression. By contrast, a high HHLA2 level predicts better survival in gastric cancer[18] and pancreatic ductal adenocarcinoma [12, 25]. Some researches have demonstrated that HHLA2 promotes T-lymphocyte proliferation and cytokine release when interacting with the TMIGD2 receptor, which suggests that HHLA2 and TMIGD2 function through a co-stimulatory pathway to stimulate T cells to eliminate cancer cells [10, 12].

Although the clinical significance of HHLA2 in some solid tumours has been clarified in recent years, the clinical significance and function of HHLA2 in ovarian cancer remains unclear. In this study, HHLA2 is more expressed in normal ovarian tissue than in cancerous tissue and high HHLA2 could serve as an independent prognostic factor for ovarian cancer, indicating that HHLA2 expression is negatively correlated with the tumorigenesis and progression of ovarian cancer. The study results also suggest that HHLA2 is expressed in well-differentiated tumours, suggesting that HHLA2 is an indicator of lower malignant tumour behaviour. We also observed a significant positive association between the CD8^+^ T-cell density and HHLA2 expression levels. The CD8^+^ TIL count in the HHLA2-positive group was increased compared with that in the HHLA2-negative group. This finding is consistent with the results in pancreatic cancer [25]. Durable anti-tumour immunity was most strongly correlated with increased numbers of CD8^+^ T cells, and the T-cell infiltration status might be affected by several factors [25]. Therefore, HHLA2 may influence T cell infiltration as a co-stimulatory molecule in ovarian cancer. Ovarian cancer cells may escape activated T-cell attack through the loss of HHLA2 protein expression. Additionally, we found no correlation between HHLA2 expression and PD-L1 and B7x expression in ovarian cancer, indicating that HHLA2 was unlikely to be co-expressed with PD-L1 or B7x. This finding was consistent with a previous finding of limited co-expression between HHLA2 and PD-L1 but not with the result of common co-expression between HHLA2 and B7x in lung cancer [11]. Because the latter study used immunohistochemistry to examine the relationship among HHLA2, PD-L1, and B7x, different research approaches and different types of tumour may partly contribute to the discrepancy.

Beyond that, we also elucidate the cell-intrinsic effects of HHLA2 in ovarian cancer cells. As an immune checkpoint molecule, no previous study has focused on the cell-intrinsic effect of HHLA2. Prior studies have reported that, in addition to its immunosuppressive function, immune checkpoint ligand like PD-L1 can regulate biological processes, including tumour growth, glucose metabolism, and autophagy, in an immune-independent manner [26]. In our study, the MTT assay and EdU staining assay showed that HHLA2-overexpressing ovarian cancer cells exhibited decreased proliferation ability compared with the normal groups, indicating that HHLA2 can inhibit the proliferation of ovarian cancer cells independent of an immune environment. Therefore, HHLA2 might affect malignant phenotypes such as tumour proliferation by exerting its tumour-intrinsic effects.

It is relatively common for members of the B7 family to have dual functions depending on the immune environment, as well as their interaction with different receptors or receptor engagement or blockade [4]. TMIGD2 is a costimulatory receptor that interacts with HHLA2 on T cells [13]. However, TMIGD2 was reported to be expressed on naïve T cells but not on other immune cells [13], and T-cell infiltration in the tumour sites comprises T effector cells derived from naïve T cells activated by antigen-presenting cells in the lymph node [27]. Thus, the receptors that can interact with HHLA2 expressed in ovarian cancer cells may not be TMIGD2. HHLA2 may stimulate T effector cells (CD8^+^ T cells) through different receptors in ovarian cancer, leading to the killing effect of CD8^+^ T cells in ovarian cancer. Future studies are needed to identify the co-stimulatory receptors on T cells infiltrated in ovarian cancer. In addition, we also find that HHLA2 inhibited ovarian cancer cells proliferation without the affecting immune cells in vitro. Because of the limit present research results, ovarian cancer proliferation under the impact of immune cells, such as CD8^+^ T cells, is still unclear. More studies are needed to explore their correlation in our future research.

## Conclusions

For the first time, HHLA2 expression in epithelial ovarian cancer tissue and its clinical significance were examined. We found that HHLA2 positivity is less common in epithelial ovarian cancer than in normal ovarian and fallopian tube epithelium, and high HHLA2 expression is an indicator of lower malignant tumour behaviour and a better prognosis in ovarian cancer. Tumour-infiltrating CD8^+^ T cells were more numerous in HHLA2-positive ovarian cancer than in HHLA2-negative ovarian cancer. Additionally, upregulated HHLA2 can inhibit the proliferation of ovarian cancer cells, supporting our findings that high HHLA2 expression is a favourable predictor for patient survival in ovarian cancer. Taken together, we provide evidence that HHLA2 may participate in cancer immune evacuation and cancer genesis. Our findings offer an alternative hypothesis: HHLA2 may act as a co-stimulatory ligand in ovarian cancer, and the absence of HHLA2 expression may contribute to the establishment of an immunosuppressive tumour microenvironment and tumour progression. Therefore, HHLA2 or its receptors may represent attractive targets for ovarian cancer patients. Future studies to determine the biological function of HHLA2 in ovarian cancer are warranted to further elucidate their contributions to tumour progression.

## Data Availability

All have been shown in the manuscript.
